# The Role of Mood Stabilizers and Antipsychotics in the Treatment of Borderline Personality Disorder

**DOI:** 10.1192/j.eurpsy.2025.548

**Published:** 2025-08-26

**Authors:** J. F. Barbosa, D. M. Silva, B. Leal, A. Lourenço, A. C. Nunes, M. A. Silva, C. Oliveira, M. Nascimento

**Affiliations:** 1 Hospital Júlio de Matos, ULS São José, Lisbon, Portugal

## Abstract

**Introduction:**

The treatment of Borderline Personality Disorder (BPD) presents a clinical challenge in many ways, as the current recommended psychotherapies are often insufficient or unavailable. As of today, no pharmacological treatment has been approved by regulatory agencies for the treatment of BPD, even though up to 96% of these patients receive at least one psychotropic medication. Some professional societies cautiously recommend the off-label and symptom-targeted use of psychotropic agents as part of a multimodal approach, whereas others recommend its use only in the event of an acute crisis.

**Objectives:**

Conduct a non systematic review of literature regarding the efficacy of mood stabilizers (MS) and antipsychotics (AP) in the treatment of patients with BPD.

**Methods:**

A search in the PubMed database was performed with the terms *borderline*, *behaviour* and *mood stabilizer* or *antipsychotic* or *pharmacological*, filtered for reviews, systematic reviews and meta-analysis over the last 20 years.

**Results:**

The efficacy of pharmacotherapies for the treatment of BPD is limited to improvement of individual symptoms but not of global functioning nor the severity of the condition overall, although the evidence is of very low certainty. For affective dysregulation and impulsive-behavioural dyscontrol, the highest efficacy emerged for MS, as AP shows a lower yet significant effect size. Both drug classes seem to improve symptoms of anger, with evidence suggesting a much larger (and significant) effect-size for aripiprazole compared to other AP. For cognitive-perceptual symptoms, only AP proved to be effective, showing higher effect-size in longer trials, which suggests their slowly progressive efficacy on this symptom dimension. Although many studies suggest a superior anti-suicide effect of clozapine in schizophrenia, the evidence is very uncertain about the effect of any medication compared with placebo on self-harm and suicide-related outcomes in patients with BPD, indicating little to no effect. There appears to be no significant difference between pharmacotherapy and placebo in terms of dropout rates, but there is insufficient data regarding drug tolerability in these patients.

**Conclusions:**

In congruence with some clinical practice guidelines, pharmacotherapy can be used to target specific core-symptoms on BPD, even though evidence on its efficacy is of very low certainty and limited to the improvement of individual symptoms but not the overall condition. Mood stabilizers and antipsychotics can have a positive effect on affective dysregulation, anger and impulsive-behavioural dyscontrol, and antipsychotics proved to be effective for cognitive-perceptual symptoms.

**Disclosure of Interest:**

None Declared

